# Improved Prediction of Ovarian Cancer Using Ensemble Classifier and Shaply Explainable AI

**DOI:** 10.3390/cancers15245793

**Published:** 2023-12-11

**Authors:** Nihal Abuzinadah, Sarath Kumar Posa, Aisha Ahmed Alarfaj, Ebtisam Abdullah Alabdulqader, Muhammad Umer, Tai-Hoon Kim, Shtwai Alsubai, Imran Ashraf

**Affiliations:** 1 Faculty of Computer Science and Information Technology, King Abdulaziz University, P.O. Box 80200, Jeddah 21589, Saudi Arabia; nabuznadah@kau.edu.sa; 2 Department of Information Science, University of Arkansas at Little Rock, Little Rock, AR 72204, USA; sharathonstream@gmail.com; 3 Department of Information Systems, College of Computer and Information Sciences, Princess Nourah bint Abdulrahman University, P.O. Box 84428, Riyadh 11671, Saudi Arabia; aiaalarfaj@pnu.edu.sa; 4 Department of Information Technology, College of Computer and Information Sciences, King Saud University, Riyadh 12372, Saudi Arabia; eabdulqader@ksu.edu.sa; 5 Department of Computer Science & Information Technology, The Islamia University of Bahawalpur, Bahawalpur 63100, Pakistan; umersabir1996@gmail.com; 6 School of Electrical and Computer Engineering, Yeosu Campus, Chonnam National University, 50, Daehak-ro, Yeosu-si 59626, Jeollanam-do, Republic of Korea; 7 Department of Computer Science, College of Computer Engineering and Sciences, Prince Sattam bin Abdulaziz University, P.O. Box 151, Al-Kharj 11942, Saudi Arabia; sa.alsubai@psau.edu.sa; 8 Department of Information and Communication Engineering, Yeungnam University, Gyeongsan 38541, Republic of Korea

**Keywords:** ovarian cancer detection, explainable AI, ensemble learning, bagging and boosting

## Abstract

**Simple Summary:**

Ovarian cancer is one of leading cause of death among women and early detection is important for timely treatment. For its detection at early stages, machine learning can be significantly important to speed up the screening and provide more accurate results. A stacked model is designed in this study, combining the strengths of mutliple models to obtain better accuracy compared to existing models. With a 96.87% accuracy, the model proves to be robust and accurate. The use of explainable AI elaborates on the importance of various appropriate features to enhance cancer detection accuracy in this regard.

**Abstract:**

The importance of detecting and preventing ovarian cancer is of utmost significance for women’s overall health and wellness. Referred to as the “silent killer,” ovarian cancer exhibits inconspicuous symptoms during its initial phases, posing a challenge for timely identification. Identification of ovarian cancer during its advanced stages significantly diminishes the likelihood of effective treatment and survival. Regular screenings, such as pelvic exams, ultrasound, and blood tests for specific biomarkers, are essential tools for detecting the disease in its early, more treatable stages. This research makes use of the Soochow University ovarian cancer dataset, containing 50 features for the accurate detection of ovarian cancer. The proposed predictive model makes use of a stacked ensemble model, merging the strengths of bagging and boosting classifiers, and aims to enhance predictive accuracy and reliability. This combination harnesses the benefits of variance reduction and improved generalization, contributing to superior ovarian cancer prediction outcomes. The proposed model gives 96.87% accuracy, which is currently the highest model result obtained on this dataset so far using all features. Moreover, the outcomes are elucidated utilizing the explainable artificial intelligence method referred to as SHAPly. The excellence of the suggested model is demonstrated through a comparison of its performance with that of other cutting-edge models.

## 1. Introduction

Ovarian cancer is currently one of the most fatal cancers among women. It was diagnosed in 295,414 women in 2018, while there were approximately 184,799 deaths globally. The exuberating mortality rate is because of poor diagnosis at the initial stage due to the lack of noticeable symptoms, resulting in poorer long-term survival prospects [1]. Despite the fact that ovarian cancers usually respond well to chemotherapy, particularly with taxane/platinum treatment, recurrence rates within 5 years for individuals with advanced disease still range from 60% to 80% [2]. According to GLOBOCAN projections, there will be an anticipated 56% surge in ovarian cancer incidence globally by 2050, with a significant 75% of cases being diagnosed in the later stages [3]. A large proportion (approximately 75%) of cases arise in post-menopausal individuals, with an occurrence rate of 40 per 100,000 per annum in patients aged 50 and above. Timely identification of this condition substantially enhances the survival rate, increasing it from 3% (at Stage IV) to 90% (at Stage I) over a span of five years [4]. The established benchmark for diagnosing and categorizing ovarian cancer is histopathological assessment, which discerns various histological variations. Accurate interpretation of cellular morphology is crucial for determining the various ovarian cancer types and guiding treatment planning [5]. Qualified pathologists with expertise in ovarian tumors are the most qualified for undertaking this responsibility. Nonetheless, instances of inconsistencies among different observers in their grading assessments have been documented. These variations in interpreting histopathology can result in imprecise prognostic projections, less than optimal therapeutic approaches, and a deterioration in the quality of life for patients [6].

Gynecologists often face the challenge of diagnosing whether a patient’s pelvic masses are malignant and possibly indicative of tumors [7]. There are multiple ways of detection of ovary cancers [8,9,10]. While definite methods such as helical computed tomography (CT) scanning [11] and sonography are employed to differentiate malignant non-gynecologic and benign conditions, detecting carbohydrate antigen 72-4 (CA72-4), carbohydrate antigen 125 (CA125) [7], and human epididymis protein 4 (HE4) as cancer biomarkers plays a crucial role in distinguishing female pelvic masses [12]. In the same way, cervical cancer detection has multiple way [13,14,15]. Several studies have investigated the differentiating efficacy of these biomarkers of benign tumors from ovarian cancer. In one study, Moore et al. compared the risk of ovarian malignancy algorithm (ROMA) and risk of malignancy index (RMI) algorithms for 457 patients to predict epithelial ovarian cancer. The authors found that ROMA exhibited higher sensitivity in identifying epithelial ovarian cancer patients compared to RMI [16]. The study by Anton et al. evaluated the sensitivity of ROMA, CA125, RMI, and HE4 in 128 patients and witnessed the highest sensitivity of HE4 in assessing malignant ovarian tumors [17]. Additionally, for predicting the progression of ovarian cancer, a multi-marker linear model was developed by Zhang et al. by incorporating HE4, estradiol, progesterone, and CA125 [18]. The model aimed to provide insights into the progression of the disease.

Ovarian cancer is a significant health concern due to its high mortality rate and impact on women’s health. Early detection is challenging because symptoms often appear late, effective screening tests are lacking, and there is a lack of awareness. The disease’s heterogeneity and limited biomarkers further complicate diagnosis. Overcoming these challenges is essential for improving outcomes and reducing the burden of ovarian cancer. Predicting the risk of developing ovarian cancer is desirable, but an initial cancer diagnosis is crucial, especially since effective early detection methods are still under development. Cancer diagnosis can be framed as a classification challenge, where the goal is to build a robust classifier to identify positive cases based on biomarkers. The effectiveness of a classifier can be assessed using metrics such as sensitivity and specificity. In sensitive medical contexts, it is often more important to understand how a model works than to achieve the highest possible accuracy. This is because doctors need to be able to trust the models they use to make decisions about patient care. Therefore, it is important to choose the right model and analytical approach for the task at hand. This will help to ensure that the results are both understandable and accurate.

The ability to classify and the degree of model interpretability are influenced by the nature of the data. For data with relatively simple patterns, simpler machine-learning models may be sufficient. Complex models may not be necessary and could lead to overfitting [19]. In such cases, analysis techniques such as Shapley Additive Explanation (SHAP) can be used to understand complex models and guide the selection of a more suitable model [20]. To address the significant health challenge of ovarian cancer and the need for early biomarker discovery, a study [21] presented a best-practice framework that combined machine learning (ML) and explainable artificial intelligence (XAI) techniques. Using SHAP values, the study demonstrated the potential for improved accuracy and utility in biomarker validation tasks.

The primary goal of this research is to develop a system capable of accurately categorizing ovarian cancer into benign and malignant. The intention is to organize cancer-related data in a manner that simplifies treatment access for patients and reduces associated risks. To achieve this, computer-aided diagnosis (CAD) methods will be utilized to assist physicians and pathologists in analyzing medical images more effectively [22,23]. Machine-learning (ML) algorithms, using innovative approaches, hold great potential for predicting disease progression and diagnosing malignancy. Despite numerous studies, the current accuracy ratings of ovarian cancer diagnosis are not satisfactory, leaving room for improvement. This proposed method introduces a novel aspect to enhance the reliability of clinical assessment, benefiting patients and healthcare professionals [24,25,26].

Predominantly, existing approaches for ovarian cancer rely on single machine learning or deep-learning algorithms and offer limited accuracy and generalizability. In addition, the major focus of existing studies is on the optimization of the model, and the feature engineering part is not very well investigated. The current study, on the other hand, proposes a stacked ensemble model to overcome the limited accuracy offered by individual models. The objective is to combine the strengths of bagging and boosting classifiers to improve predictive accuracy and reliability. By combining these two techniques, this research exploits the benefits of variance reduction and improved generalization, leading to superior ovarian cancer prediction outcomes. The key areas of our research are as follows:

- This study introduces a comprehensive framework designed for the identification of ovarian cancer through the utilization of feature-based data. The prediction of ovarian cancer is carried out through the application of an ensemble approach involving bagging and boosting classifiers.
- For a performance comparison, this study employs a diverse set of machine-learning models, encompassing extreme gradient boosting (XGB), random forest (RF), stochastic gradient descent (SGD), K-nearest neighbor (KNN), extra-trees classifier (ETC), and gradient boosting machine (GBM) models. Moreover, to facilitate comparison, the effectiveness of the proposed system is compared with several established cutting-edge techniques, utilizing commonly recognized evaluation metrics such as accuracy, precision, recall, and F1 score.
- The results obtained are further explained using the explainable AI technique Shapley additive explanations (SHAP) to show the contribution of each feature in the prediction.

The paper’s organization is as follows: In Section 2, we perform an extensive review of existing literature related to the detection of ovarian cancer using CAD. Section 3 outlines the materials and methodologies employed in this research. The discussion of experimental outcomes and results is the focal point of Section 4. Finally, Section 5 provides the conclusions and future research directions.

## 2. Related Work

This section discusses various methods for categorizing ovarian cancer, based on cell type. Accurate identification of the ovarian cancer type is crucial for creating personalized treatment plans for patients. Numerous studies have aimed to improve the cancer screening process and have resulted in the preclinical stage during the last decade. However, manual image analysis by expert pathologists is not consistent among different individuals, and it is also very time-consuming. Recently, ML algorithms have been vigorously used for the initial screening and diagnosis of ovarian cancer. Various methods are recommended for feature extraction from ultrasonic images and subsequent classification. This section also explores several state-of-the-art ML-based approaches for detecting ovarian cancer.

Maria et al. [27] presented a machine-learning approach for ovarian cancer classification, employing six models: linear discriminant analysis (LDA), classification and regression tree (CART), logistic regression (LR), Naive Bayes (NB), KNN, and support vector machine (SVM). Remarkably, LR, CART, and LDA achieved an impressive accuracy of 100% for ovarian cancer classification on the Kaggle dataset. Their experimentation involved using only seven biomarkers from the dataset. On the other hand, Han et al. [28] proposed an efficient machine-learning-based regularized LR model for ovarian cancer prediction. The authors identified 30 crucial features within a single unified network and utilized LR with LASSO regularization. The approach yielded an accuracy of 90.6%.

One study [29] introduced a group-penalized LR model to predict ovarian cancer. The authors combined group SCAD/LASSO/MCP-penalized LR with an ML model to enhance accuracy. The study utilized 46 features from the dataset, organized into 11 distinct groups. The penalized LR process identified three groups, with Group MCP achieving the highest accuracy of 93.33%. On the other hand, Ziyambe et al. [30] proposed an efficient deep-learning-based system for diagnosing ovarian cancer. The authors trained a convolutional neural network (CNN) model on a histopathological image dataset, achieving an accuracy score of 94%. In the study, the authors also compared the performance of deep- and transfer-learning models for this task.

Kalaiyarasi et al. introduced a state-of-the-art system for performance analysis of machine-learning models using the microarray gene data of ovarian cancer [31]. The authors utilized various techniques, such as discrete cosine transform (DCT), SDA, Hilbert transformation, fast Fourier transform (FFT), and fuzzy C-means cluster (FCM) for feature selection. The results revealed that the Gaussian mixture model with DCT features achieved an accuracy of 88% for ovarian cancer prognosis. On a similar note, Azar et al. [32] projected an ML-based system to efficiently predict the survival of ovarian cancer patients. They employed six machine-learning models and used SHAP to ascertain feature importance for clearer decision-making. The study demonstrated that the RF model attained an 88.72% accuracy. This success can be attributed to SHAP’s efficient identification of important features through extracted decision trees of the RF.

Ahamad et al. [33] presented an ML-based approach for the initial prognosis of ovarian cancer. They partitioned the dataset’s 49 features into three distinct sets and conducted experiments on these selected features. The study revealed that the ML models RF, GBM, and LGBM achieved an accuracy of 91% using the selected features. Similarly, Lu et al. [12] offered an ML-based system that employed maximum relevance–minimum redundancy (MRMR) feature selection to extract relevant dataset features. The authors utilized the ML models ROMA, DT, and LR. The results demonstrated that the LR model, using the top 10 features, achieved a remarkable accuracy of 97.4%.

Kasture et al. [34] introduced an enhanced deep CNN called KK-Net, which efficiently and accurately classified four different classes. The authors also conducted comparisons with multiple deep-learning and transfer-learning models for benchmarking. The study results indicate that the proposed KK-Net achieved an accuracy of 91% on the TCGA dataset. In a similar vein, Akazawa et al. [35] suggested a machine-learning-based system for ovarian cancer detection. The study revealed that the ML model XGBoost (XGB) achieved an accuracy of 80%.

Explainable AI (XAI) aims to make AI systems visible and intelligible to humans. It tries to clarify how AI makes judgments, building trust and enabling the detection of biases or errors. This openness is critical in a variety of domains to enable responsible and ethical AI implementation. Arrieta et al. [36] provided an analysis of previous studies contributing to the domain of XAI. Consiglio et al. [37] applied fuzzy rules to explain the gene expression of ovarian cancer. Lios et al. [38] predicted surgical cytoreduction in ovarian cancer and also provided a model explanation using SHAP (Copyright 2018, Scott Lundberg, Built with Sphinx and GoogleDeepMind location: Seattle, DC, USA). The authors merged advanced AI modeling with the XAI framework to elucidate the impact and relationships of features linked to a particular surgical difficulty level [39]. Huang et al. [21] performed discovery biomarkers using machine-learning models and also used XAI to explain models. Additionally, the authors conducted a feature importance analysis in the work.

A comprehensive summary of the related work is given in Table 1. Despite the results reported in existing studies, some of which were very good, several issues and limitations need further research for ovarian cancer. One study [27] utilized LR, NB, KNN, CART, NB, LDA, and SVM and used only accuracy as an evaluation metric; precision, recall, and F1 scores were not reported. In addition, no cross-validation was carried out. Similarly, ref. [28] focused only on increasing the prediction accuracy using LR with LASSO. Cross-validation and performance comparison with other models was not considered.

In the same vein, in ref. [29], experiments were performed using LR with different penalties such as LASSO, SCAD, MCP, etc., along with ANN and SVM models. No validation was carried out in this study and performance was not compared with state-of-the-art models. Similarly, in [30], experiments were performed using a single dataset and the results were reported using accuracy, precision, recall, and F1 score. However, cross-validation was not considered.

The authors utilized several models in [31] for ovarian cancer detection and ran experiments with and without selective features. Despite good results from the study, K-fold cross-validation was not carried out, thereby leaving doubts about the robustness and generalizability of the proposed approach. Azar et al. [32] utilized radiomic features for cancer detection and employed cross-validation, but the reported accuracy was low, with approximately 70% accuracy on average.

A few studies have reported very high accuracy. In particular, refs. [12,27] reported an accuracy of 100% and 97.4%, respectively. However, the accuracy of 100% shows a case of model overfit. These works utilize a few selected features from 49 features but no justification for feature selection is provided. Generalizability is questioned as K-fold cross-validation is not considered to check the overfitting. Furthermore, ref. [12] completed the missing values of the dataset using mean values. This also supports the idea of model overfitting.

## 3. Proposed Approach

**Figure 1:**
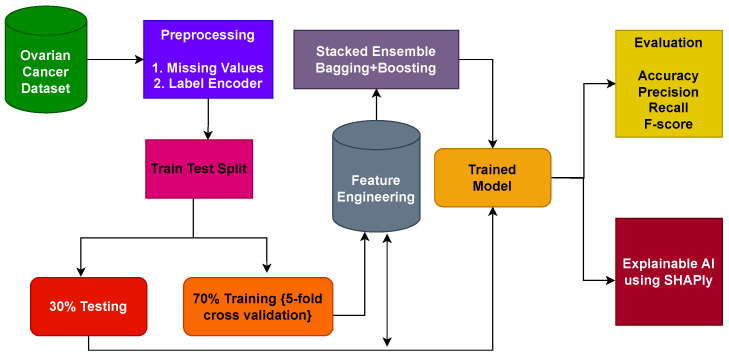
Architectural diagram of the proposed methodology indicating the steps, while arrows indicate the flow of steps.

This section provides a concise overview of the materials and methods employed in classifying ovarian cancer. It encompasses details about the dataset used, the ML models utilized for ovarian cancer detection, the proposed methodology, and the assessment parameters employed to measure the performance of the learning models. Figure 1 shows the workflow of the methodology adopted in this study.

### 3.1. Dataset

The study utilized a dataset containing 349 individuals, sourced from the Third Affiliated Hospital of Soochow University. The data were gathered over the period from July 2011 to July 2018, and it encompassed two distinct categories: a group of 178 patients diagnosed with benign ovarian tumors, and another group comprising 171 patients diagnosed with ovarian cancer (https://www.kaggle.com/datasets/saurabhshahane/predict-ovarian-cancer, accessed on 27 October 2023). In total, the dataset contained 49 features, which were obtained through pathology diagnosis. These 49 predictor variables consisted of 22 general chemical tests, 19 blood routine tests, and 6 tumor markers, including age and menopause information. All patients underwent postoperative case diagnosis, and none of them had received preoperative radiotherapy or chemotherapy. The histological diagnosis was classified based on World Health Organization criteria.

### 3.2. Machine-Learning Models

ML has shown great promise in various medical fields, including cancer prediction and diagnosis. When it comes to ovarian cancer prediction, ML algorithms can be utilized to analyze and interpret large amounts of patient data, helping to identify potential cases earlier and more accurately. The ML model’s success in ovarian cancer prediction depends upon the quality and quantity of data used for training. Access to a diverse and representative dataset is crucial for building an accurate and reliable predictive model. RF, KNN, SGD, ETC, XGB, and GBM are utilized in this study. The details of the model’s hyperparameters are shown in Table 2. We have utilized the gridsearchCV method to obtain the best hyperparameters. This function uses a list of parameters where a range for each parameter can be specified. To determine an appropriate range for each parameter, we analyzed the existing literature.

#### 3.2.1. K Nearest Neighbor

KNN is an easy-to-implement and interpret ML algorithm utilized for both regression and classification tasks. KNN is a lazy learner and leverages the entire dataset [40,41]. It compares the training data with test data and then evaluates the distance in the training dataset between the nearest neighbors. KNN can employ various distance metrics, with the Euclidean distance being the most frequently used.

(1) $$Euclidean=\sqrt{\sum_{p=1}^{n}{\left(i_{p}-j_{p}\right)}^{2}}$$

In the context of Euclidean *n*-space, *i* and *j* represent two points and “$i_{p}-j_{p}$” denotes the Euclidean vectors starting from the origin of the space. Specifically, it represents the difference between the coordinates of the points i and j in the Euclidean *n*-space.

#### 3.2.2. Random Forest

RF is another model based on decision tree (DT), consisting of an ensemble of multiple DTs. RF is applicable for both classification and regression tasks and demonstrates strong performance on datasets with nonlinearity and class imbalance [42,43]. RF combines the predictions of multiple DTs using a majority voting approach. Each DT in the RF independently predicts the outcome for the test data, and the final prediction of the RF is determined by selecting the class that is most frequently predicted by the individual DTs.

(2) $$rf=modeDT_{1},DT_{2},DT_{3},\cdots ,DT_{n}$$

(3) $$rf=mode\sum_{i=1}^{N}DT_{n}$$

where $DT_{1}$, $DT_{2}$, and $DT_{3}$ represent individual decision trees within the ensemble. The variable *n* corresponds to tree numbers in the random forest.

#### 3.2.3. Stochastic Gradient Descent

SGDC is based on the principles of LR and SVM classifiers [44,45]. SGDC utilizes the convex loss function from LR, making it a robust classifier. It particularly excels in multiclass classification tasks, employing the One-versus-All (OvA) approach to combine multiple classifiers. One of the notable advantages of SGDC is its efficiency in handling large datasets, as it processes a single example per iteration. Furthermore, SGDC is relatively easy to implement and comprehend due to its reliance on regression techniques. To achieve optimal results, appropriate parameter tuning is crucial for SGDC. Additionally, it is important to note that feature scaling plays a significant role in the sensitivity of SGDC.

#### 3.2.4. Extra Tree Classifier

The ETC algorithm is comparable to the RF technique but builds trees differently [46,47]. Unlike RF, ETC uses original data to construct trees rather than using samples from bootstrap data. In ETC, decision-making is based on random data sampling from the K-best characteristics. The Gini index is used to find the top feature for dividing the tree. ETC and RF are regarded as equivalent since both are ensemble learning models used for categorization. The main distinction between ETC and RF lies in how the trees are constructed within the forests. In ETC, K features’ random samples are drawn from feature collection randomly and distributed to each tree’s test node.

#### 3.2.5. Gradient Boosting Machine

GBM is a strong learning classifier made up of multiple weaker classifiers. GBM is based on DT and builds separate trees, resulting in a longer execution time. The algorithm has been improved through tweaks, specifically the probability approximately correct (PAC) learning algorithm, which enhances its performance [48,49]. GBM handles missing values effectively and hence performs well on raw data. GBM requires a segregated loss mechanism to work. While regression methods commonly use logarithmic loss, classification algorithms can also utilize it. The advantage of GBM is that it can employ any differentiable loss function instead of creating a new one for each boosting iteration. Several hyper-parameters in GBM must be tweaked to attain good accuracy. Setting “n” to 100, for example, shows 100 trees contributing to the forecast. Averaging all 100 decision trees projections is needed to arrive at the final projections. The “max_depth” option can be used to limit the maximum depth of 60 levels.

#### 3.2.6. Extreme Gradient Boost

This is a famous ML algorithm designed for regression and classification tasks. A boosting technique combining predictions of numerous weak learners (typically decision trees) is employed to create a stronger ensemble model [50,51]. XGBoost optimizes a differentiable loss function using gradient boosting, which emphasizes model accuracy and efficiency. Its success lies in handling complex datasets, avoiding overfitting through regularization, and providing excellent performance in various domains, making it a top choice for many data scientists and machine-learning practitioners.

### 3.3. Proposed Framework

**Figure 2:**
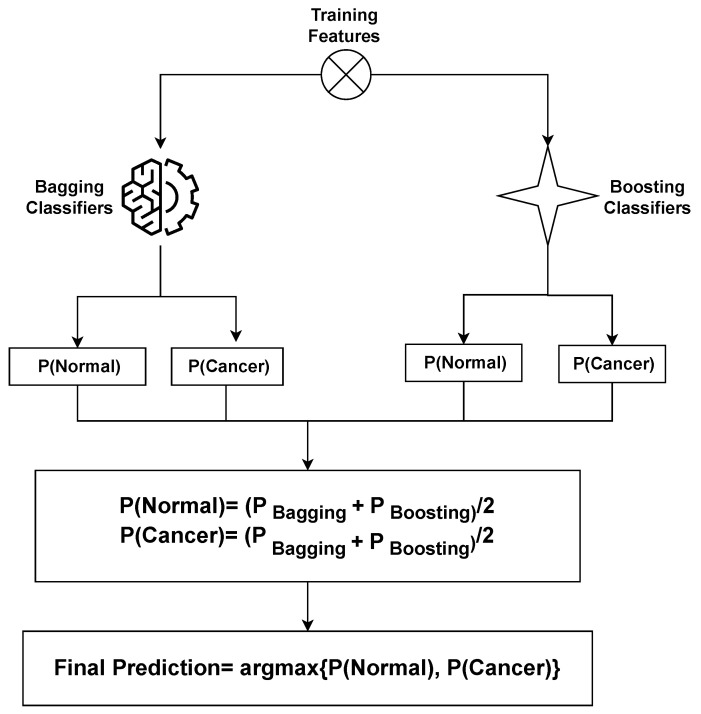
Architecture of the proposed stacked ensemble that combines bagging and boosting classifiers to increase the classification accuracy of ovarian cancer.

This section outlines the arrangement of the proposed methodology and its individual components as implemented within the experiment. Figure 2 presents a detailed depiction of the structure of the proposed framework. The proposed approach comprises two phases. In the first phase, all learning algorithms are implemented and the proposed stacked ensemble model, a fusion of bagging and boosting classifiers, undergoes training on the dataset. Transitioning to the second phase, the SHAP explainable AI technique is utilized. This technique provides insights into the contribution of features toward predictions. SHAP methodically unveils the extent to which each feature participates in the ovarian cancer prediction process.

The rationale behind adopting this stacking approach lies in harnessing the expertise of bagging and boosting classifiers. The objective is to craft a more resilient and precise framework that facilitates the early detection of ovarian cancer. In the medical domain, prediction accuracy holds immense significance. The application of the SHAP technique aids in portraying the final prediction in relation to features, offering a comprehensive explanation of the model’s performance. The complete algorithm of the voting classifier is shown in Algorithm 1.
**Algorithm 1:** Voting classifier with bagging and boosting models
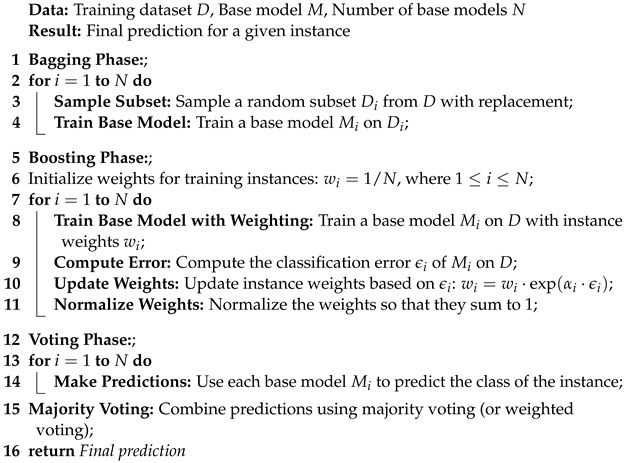


#### Evaluation Parameters

This study employs multiple assessment metrics to evaluate classifier performance. These metrics include F1 score, accuracy, recall, and precision, which are computed based on the values of false negatives (FN), true positives (TP), true negatives (TN), and false positives (FP).

Accuracy shows the overall correctness of the model’s predictions using the ratio of the truly classified samples (both positive and negative) to the total samples in the dataset

(4) $$Accuracy=\frac{TP+TN}{TP+TN+FP+FN}$$

Recall, also known as a delicacy or true positive rate, assesses the classifier’s ability to correctly identify positive samples within a particular class. The recall is calculated using

(5) $$Recall=\frac{TP}{TP+FN}$$

Precision quantifies the percentage of correctly identified positive samples among all the samples predicted as positive. It is determined using

(6) $$Precision=\frac{TP}{TP+FP}$$

The F1 score is utilized where data class imbalance occurs, combining both precision and recall into a single score. It is given by

(7) $$F1-Score=2\times \frac{precision\times recall}{precision+recall}$$

These evaluation metrics help in comprehensively evaluating the classifiers performance, taking into account different aspects of their predictions, such as false negatives, false positives, true positives, and true negatives.

## 4. Results and Discussions

This study employs a variety of ML models for ovarian cancer detection. The dataset is divided into training and testing sets in the ratio of 70:30, a widely adopted practice in various classification studies to mitigate overfitting. The assessment of performance involves diverse evaluation metrics tailored for the ML classifiers. All experimental procedures are executed within a Python, ver. 3.12.0, environment, utilizing various libraries, on a Dell PowerEdge T430 GPU (Chongqing, China) with 2 GB capacity, housing 2× Intel Xeon 8 Cores operating at 2.4 GHz, and equipped with 32 GB of DDR4 RAM.

### 4.1. Results of the Machine-Learning Models

A comprehensive evaluation of supervised ML classifiers was conducted on the complete set of features within the ovarian cancer dataset. This study encompassed a range of regression-based, tree-based, and statistical-based models for ovarian cancer prediction. The performance assessment of all ML models using the entire feature set is presented in Table 3. The measurements used to evaluate the performance of the machine-learning models presented in Table 3 include accuracy, precision, recall, and F1 score. In Table 3, the accuracy values range from 77.94% to 87.14%. Higher accuracy indicates a better overall performance of the model; however, accuracy alone is not enough to highlight a model’s performance. The precision values range between 77.43% and 87.58%. Higher precision indicates fewer false positives. The recall values range from 78.36% to 87.56%. Higher recall indicates fewer false negatives. F1 score values range from 77.98% to 87.22%. A higher F1 score indicates a better balance between precision and recall.

As per the results, the ETC classifier achieved 86.89% accuracy, with an 86.52% score for recall, precision, and F1 score alike. The RF ensemble model attained 85.66% accuracy and 86.22% F1 score. The GBM classifier outperformed the rest using all features except for recall score and obtained 87.14% accuracy, 87.53% recall, 87.58% precision, and 87.19% F1 score. The KNN is the worst classifier for ovarian cancer prediction with 78.36% recall, 77.94% accuracy, 77.43% precision, and 77.98% F1 score. Performance comparison of all models is presented in the figure below.

### 4.2. Results of Ensemble Models

The evaluation of various models and ensemble techniques for ovarian cancer classification provided insightful performance metrics, as summarized in Table 4. Notably, this study employed two baseline models (SGD and KNN), two bagging classifiers (ETC and RF), and two boosting classifiers (GBM and XGB), followed by an investigation into their ensemble configurations.

The ensemble models results demonstrate diverse performance across different metrics, shedding light on the efficacy of these methodologies for ovarian cancer prediction. It is evident that the ensemble of boosting models (GBM + XGB) exhibited superior performance compared to both the baseline and bagging classifier ensembles. This ensemble achieved an accuracy of 88.24%, a precision score of 88.68%, a recall of 88.49%, and an F1 score of 88.69%. These metrics collectively indicate the robustness and efficiency of the boosting ensemble in accurately predicting ovarian cancer.

In contrast, the baseline classifier ensemble (SGD + KNN) showcased commendable performance, achieving an accuracy of 85.94%. While this ensemble demonstrated strong predictive capabilities, its performance metrics fell slightly behind those of the boosting ensemble. Interestingly, the bagging classifiers ensemble (ETC + RF) yielded the least favorable performance among the ensemble models evaluated in this study, achieving an accuracy of 84.93%. Despite its utilization of ensemble techniques, the bagging classifier ensemble marginally lagged behind both the baseline and boosting ensembles in terms of predictive accuracy, precision, recall, and F1 score.

These results underscore the significance of selecting appropriate ensemble methodologies for improving ovarian cancer prediction. The notable performance of the boosting ensemble advocates for its adoption in future predictive models for ovarian cancer, owing to its superior accuracy and precision in identifying and classifying cases. Additionally, the comparative analysis among these ensemble strategies highlights the nuanced differences in their predictive capabilities, aiding researchers and practitioners in making informed decisions when designing and deploying predictive models in medical contexts.

### 4.3. Results of Proposed Stacked Ensemble Model

In the realm of ovarian cancer classification, this study introduced a novel approach centered around a stacking classifier system designed to integrate the strengths of ensemble bagging and boosting techniques while leveraging selective features identified through the application of the SHAP method for comprehensive feature analysis. The results attained through the proposed ensemble model are summarized in Table 5.

The experimental outcomes notably present the remarkable performance of the proposed stacking ensemble model in the context of ovarian cancer prediction. The stacking classifier demonstrated exceptional accuracy, achieving an impressive score of 96.87%. This substantial accuracy level signifies the model’s ability to effectively discern and classify ovarian cancer cases, showcasing a notably higher accuracy compared to the previously evaluated models.

Beyond accuracy, the proposed stacking classifier exhibited superior performance across other critical evaluation metrics. With a precision score of 98.84%, recall of 98.92%, and an F1 score of 98.88%, this ensemble model consistently outperformed the other learning models considered in this study. These metrics collectively portray the stacking classifier’s capability to not only accurately identify positive instances of ovarian cancer but also minimize false positives and negatives, indicating its robustness in both sensitivity and specificity. The outstanding performance of the proposed ensemble model underscores its potential as a highly effective tool for ovarian cancer classification. Achieving such high precision and recall rates is pivotal in the medical domain, where accurately identifying and diagnosing conditions such as ovarian cancer can significantly impact patient outcomes and treatment strategies.

The introduction of the stacking classifier, incorporating selective features identified through SHAP analysis, showcases a promising avenue for enhancing predictive models in medical diagnostics. The substantial performance improvements demonstrated by this ensemble model warrant further exploration and consideration in clinical settings, potentially contributing to more accurate and reliable early detection of ovarian cancer. The study’s findings suggest that the proposed ensemble model holds considerable promise for practical implementation, potentially revolutionizing the landscape of ovarian cancer diagnostics with its exceptional predictive capabilities.

### 4.4. Shapley Additive Explanations

It might be difficult to understand the connections between inputs and outputs because ML models are thought of as black-box algorithms. When working with labeled data, in particular, this lack of interpretability results in a restricted understanding of the significance of features in supervised learning on both a global and a local scale. A recent development, the SHAP technique, resolves this problem by offering a quantitative method to gauge model interpretability. The significance of elements inside the model may now be better understood thanks to this advancement, which was first made by Lee and Lundberg in 2017 and furthered by Lundberg et al. in 2018 [52,53].

The linear additive feature attribute method used by SHAP, which is based on ideas from cooperative game theory, is used to describe complex models. In this method, an importance value is imparted to each attribute on how it affects the model’s forecasting depending on feature presence or absence during SHAP estimation. To make the operation of complex models more understandable, this approach of explanation offers a simpler model. The use of the linear additive feature attribute technique and principles from cooperative game theory is detailed in publications by Lee and Lundberg (2017), furthered by Lundberg et al. (2020) [52,53].

(8) $$f\left(a\right)=g\left(a^{\prime }\right)=\phi_{0}+\sum_{j=1}^{j}\phi_{j}a_{j}^{\prime }$$

The original ML model that we are explaining, in this case, is marked as (a), and the more straightforward explanation model is labeled as g($a^{\prime }$). $a_{j}^{\prime }$, where *j* is a simplified input seismic attribute number, denotes these attributes. All conceivable input orderings are used to calculate the SHAP values, indicated as j. The presence or absence of a certain seismic attribute is specified using an input vector that, during estimation, is called $a_{j}^{\prime }$. Last but not least, $\phi_{0}$ denotes the model prediction when none of the qualities are taken into account during the estimate. The complete feature importance calculated using SHAPly in descending order is shown in Table 6.

SHAP analysis underscores the importance of features in relation to predicting ovarian cancer. Although SHAP feature importance outperforms traditional methods, using it alone provides only limited extra insights. Beeswarm plots offer a more detailed and information-packed representation of SHAP values, revealing the relative significance of features and their intricate relationships with the predicted outcome.

SHAP beeswarm plots are a visualization tool used to explain the output of machine-learning models by showing how each feature contributes to individual predictions. They combine aspects of beeswarm plots and summary plots to provide a clear view of feature importance. The values and colors in SHAP beeswarm plots can be broken down as follows:

- Values (X-Axis): The X-axis of a SHAP beeswarm plot represents the SHAP values associated with each feature for each data point in your dataset. SHAP values quantify the impact of each feature on the model’s output for a specific prediction. These values can be both positive and negative.
- Positive SHAP Values: These indicate that the feature contributes positively to increasing the model’s output for that particular prediction. For example, a high positive SHAP value for a feature might mean that an above-average value of that feature is pushing the prediction higher. In our case, the future HE4 has the highest number of positive samples.
- Negative SHAP Values: Conversely, negative SHAP values suggest that the feature contributes negatively to the model’s output for that prediction. A high negative SHAP value for a feature implies that an above-average value of that feature is pushing the prediction lower. Again, the future HE4 has the highest number of negative samples. This clearly means that the samples of feature HE4 play a vital role in any class prediction. They are not neutral or misleading.
- Colors (Y-Axis): The Y-axis of a SHAP beeswarm plot represents the features themselves. Each point on the Y-axis corresponds to a feature in the dataset. The color of each point is used to indicate the feature’s value.
- Color Shading: The intensity of the color (e.g., darker (red) or lighter (blue) shades) can also provide additional information. A darker shade of color indicates a stronger or more influential feature value. In our case, the samples of feature HE4 are highest in both class predictions but the samples are more influential in predicting the negative class due to stronger red shades.

By examining a SHAP beeswarm plot, one can gain insights into which features are driving individual predictions and whether they are having a positive or negative impact. This visualization is especially useful for understanding how a model makes decisions on a per-instance basis and for identifying which features are most important for specific predictions. It is a powerful tool for model interpretability and can help one diagnose model behavior and make informed decisions based on model outputs.

**Figure 3:**
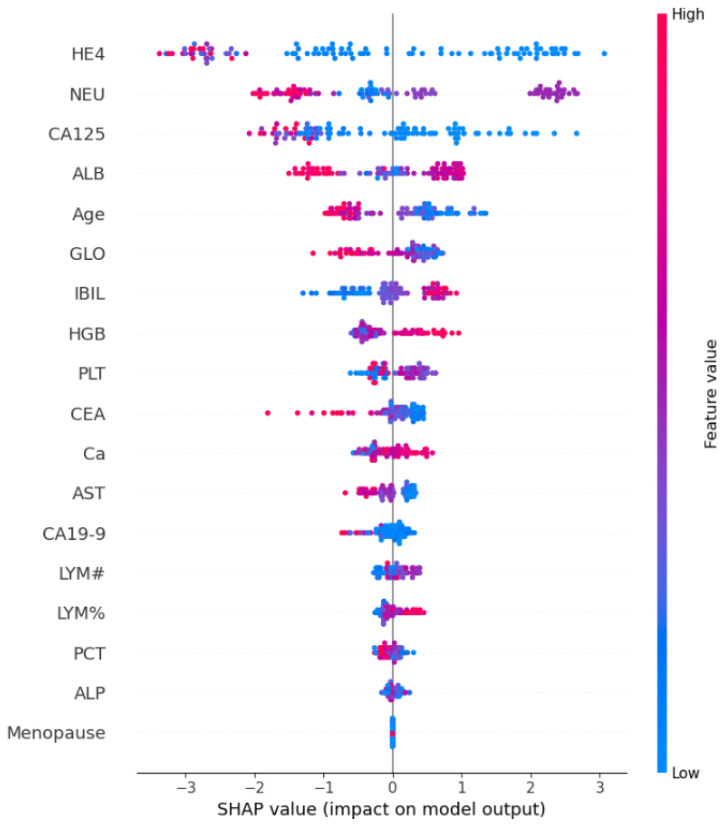
Graphical representation of SHAP feature importance, where color intensity indicates a feature importance; red color indicates higher feature importance while blue color shows less importance of a feature.

The SHAP summary demonstrates the contribution of each feature to every individual case in the dataset. The combined effects of feature contributions and the bias element result in the model’s initial prediction, which represents the prediction before the inverse link function is applied. The visual representation of SHAP feature contributions can be seen in Figure 3. Noteworthy is the observation that HE4 and NEU stand out as crucial features, with a significant number of cases. In contrast, the majority of other features are predominantly associated with predicting the presence of ovarian cancer.

The SHAP explanation provides insights into how features contribute to a particular instance. The cumulative sum of feature contributions, along with the bias term, equals the model’s original prediction, representing the forecast prior to the application of the inverse link function. For complete implementation code of SHAP see Appendix A.

### 4.5. Discussion

**Figure 4:**
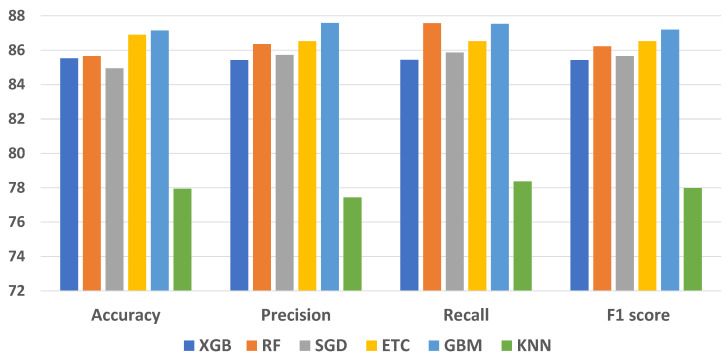
Performance comparison of machine-learning models.

**Figure 5:**
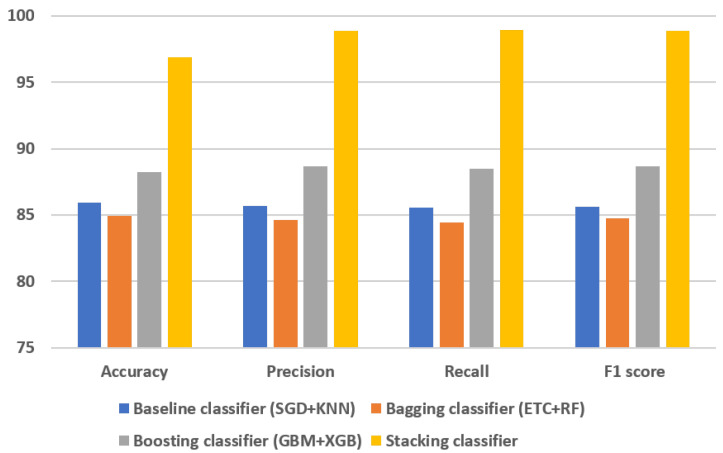
Comparison of ensemble and stacked ensemble models.

Assessing the outcomes is essential for gaining a comprehensive understanding of a model’s performance. Figure 4 presents the comparative analysis of the machine-learning models. In the evaluation, the results of individual ensemble models are compared with those of the proposed stacked ensemble model, which combines boosting and bagging classifiers. Impressively, the proposed stacked ensemble model achieved the highest level of performance, with an accuracy rate of 96.87%, as shown in Figure 5. This remarkable accuracy underscores the potential of this ensemble approach for ovarian cancer prediction.

Moreover, the intricate relationships between the variables and their corresponding SHAP values are analyzed, as visualized in Figure 3. One noteworthy observation was the elevated variable values associated with HE4, signifying that instances with high HE4 values had a pronounced negative influence on the predicted outcomes. However, it is essential to highlight a fascinating contrast: instances with elevated ALB (albumin) values were found to contribute positively to the prediction outcome. This intriguing finding suggests that the relationship between ALB values and ovarian cancer prediction is complex and multifaceted, warranting further investigation.

The proposed workflow seamlessly guides the entire process from data collection to result evaluation, integrating classification and XAI techniques. Furthermore, it presents how SHAP results can be interpreted and harnessed in conjunction with domain knowledge, enhancing the model’s interpretability and real-world applicability. The proposed approach has proven to be highly effective, particularly for the categorical datasets of biomarkers. The outstanding performance of the stacked ensemble model and the insights gleaned from SHAP analysis exemplify the potential of this methodology in the critical domain of ovarian cancer prediction.

XAI provides essential interpretability advantages in healthcare by offering clear insights into how each feature influences model predictions. In ovarian cancer prediction, this enables healthcare professionals to understand the significance of biomarkers and to prioritize assessments and builds trust in the model’s decisions. Interpretability also aids in informed decision-making, patient communication, clinical validation, and ensuring safety and accountability in healthcare AI. Overall, XAI is a critical asset for improving patient care and outcomes in the medical domain.

The improved prediction results obtained from the study hold significant implications in terms of both clinical applications and biological insights. On the clinical front, the enhanced accuracy, precision, recall, and F1 scores indicate that the ML models are adept at ovarian cancer detection, potentially leading to earlier diagnoses and better patient outcomes. The reduction in false positives and negatives, as well as the balanced F1 scores, underscores the models’ clinical utility. Moreover, the application of Shapley’s explainable AI for model interpretation provides valuable biological insights. It highlights the importance of specific features, potentially revealing key biomarkers and shedding light on the underlying biological pathways involved in ovarian cancer. This knowledge can guide future research, offering a deeper understanding of the disease’s biology, subtypes, and heterogeneity, thereby advancing the development of more targeted and effective treatments.

The proposed approach addresses the practical use, generalizability, and ethics in ovarian cancer detection. The proposed approach prioritizes model performance by achieving high accuracy and a balanced precision–recall trade-off. This improvement in prediction results makes the approach valuable in a clinical context, as it enhances the diagnostic process and reduces false positives and negatives, thereby improving patient outcomes. Shapley promotes trust and better decision-making. Rigorous validation through K-fold ensures robustness and suitability for various clinical scenarios. Comprehensive measures address ethical concerns, such as data privacy, security, and bias, aligning with healthcare equity and regulatory standards. The transparency and accessibility of model explanations to healthcare professionals and continuous maintenance solidify the approach’s responsible and ethical use in clinical practice.

The study showcases a highly accurate stacked ensemble model for ovarian cancer prediction. The SHAP analysis reveals complex relationships between biomarkers, offering valuable insights. The integrated workflow combines classification and XAI techniques effectively, enhancing model interpretability. Overall, the findings have promising implications for clinical applications and advancing our understanding of ovarian cancer biology.

### 4.6. Study Limitations

The limitations and challenges encountered during ovarian cancer detection are as follows:

- **Class distribution imbalance:** The dataset may have a disproportionate number of negative examples (i.e., samples without ovarian cancer), which can bias the model towards predicting negative results. This can lead to decreased recall and F1 scores, which are important metrics for cancer detection.
- **Limited hardware resources:** Training complex models on large datasets requires significant computing power. If sufficient hardware resources are not available, it may be necessary to use simpler models or smaller datasets, which can reduce performance.
- **Generalization to new patient populations and data sources:** Models trained on one dataset may not perform well on other datasets, especially if the datasets differ in terms of patient demographics, clinical characteristics, or image quality. It is therefore important to validate models on external datasets to assess their generalizability.

### 4.7. Performance Comparison with Existing Studies

A comprehensive comparison was conducted to evaluate the performance of the proposed model against existing state-of-the-art models, all aimed at enhancing accuracy. The comparison is shown in Table 7. These selected works are used as benchmarks to assess the effectiveness of the proposed model and highlight its advancements over existing approaches. By comparing the results of advanced models with the proposed model, this research work offers valuable insights into its superior performance in terms of accuracy improvement. We can also observe in Table 7 that some researchers [12,27] tried to simplify this problem using a simple logistic regression (LR) model with fewer features (7∼10). The issue is that logistic regression becomes easily overfitted with a small sample size, as parameter estimates can be unstable and have a high variance. In this way, logistic regression captures noise instead of true patterns. In both these research works [12,27], no regularization techniques (L1 or L2) are applied to mitigate the effect of overfitting. Selecting fewer features and a small number of samples is the main problem, which is why the results obtained are at a much higher accuracy rate. For instance, in [28] the authors employed LR with LASSO and used 30 features of the dataset, which resulted in an accuracy score of 90.6%. In [29], the MCP models were used for ovarian cancer detection, achieving an accuracy of 93.33%, but they utilized only 46 features from the dataset. Similarly, in [54] the authors applied RF for ovarian cancer using the OC Marker dataset and achieved 91% accuracy. Similarly, in [33] individual learning models such as LGBM, GBM, and LR were employed for ovarian cancer prediction, achieving an accuracy of 91% on selected features. Table 7 compares the performances of the existing studies with the proposed model. The results unequivocally demonstrate that the proposed model outperforms the existing models across various performance metrics.

We can also be observed in Table 7 that some researchers [12,27] tried to simplify this problem using a simple logistic regression (LR) model with fewer features (7∼10). The issue is that logistic regression becomes easily overfitted with a small sample size, as parameter estimates can be unstable and have a high variance. In this way, logistic regression captures noise instead of true patterns. In both these research works [12,27], no regularization techniques (L1 or L2) are applied to mitigate the effect of overfitting. Selecting fewer features and a small number of samples may lead to a higher accuracy; however, such studies lack generalizability and robustness. Despite showing high accuracy for ovarian classification, changes in the dataset and number of features would greatly affect the performance of such models. The proposed model, on the other hand, offers better accuracy with generalizability and robustness.

### 4.8. Practical Applicability and Generalizability

The proposed approach prioritizes model performance by achieving high accuracy and a balanced precision–recall trade-off. This improvement in prediction results makes the approach valuable in a clinical context, as it enhances the diagnostic process and reduces false positives and negatives, thereby improving patient outcomes. Shapley promotes trust and better decision-making. Comprehensive measures address ethical concerns, such as data privacy, security, and bias, aligning with healthcare equity and regulatory standards. The transparency and accessibility of model explanations to healthcare professionals and continuous maintenance solidify the approach’s responsible and ethical use in clinical practice. Rigorous validation through K-fold ensures robustness and suitability for various clinical scenarios.

K-fold cross-validation is utilized to guarantee the dependability of the models. Table 8 displays the outcomes of 5-fold cross-validation during the testing phase, demonstrating unequivocally that the suggested approach surpasses alternative models in accuracy, precision, recall, and F1 score. Table 9 displays the outcomes of 5-fold cross-validation during the training phase. Furthermore, the proposed approach displays minimal standard deviation, highlighting its reliability and consistency. These results indicate that the proposed approach consistently delivers strong performance across numerous folds, instilling even more confidence in its reliability and robustness.

## 5. Conclusions

Ovarian cancer is a deadly disease, but early detection and intervention can significantly improve patient outcomes. This study addresses the critical challenge of ovarian cancer prediction by developing an innovative stacked ensemble model that combines the strengths of bagging and boosting classifiers. The model leverages feature-based data to learn complex patterns associated with the disease, resulting in superior predictive performance compared to state-of-the-art methods. This research has the potential to contribute to the vital goal of improving early diagnosis and prognosis of ovarian cancer. The introduction of a stacked ensemble model, merging the strengths of bagging and boosting classifiers, aims to enhance predictive accuracy and reliability. This combination harnesses the benefits of variance reduction and improved generalization, contributing to superior ovarian cancer prediction outcomes. This study also employed XAI techniques, specifically SHAP, to understand how our predictive model works. SHAP is a powerful tool that helps us identify the most important features that contribute to a patient’s risk of ovarian cancer. This information can be used by clinicians to make more informed decisions about patient care. The research findings have significant potential for healthcare practitioners, providing them with a valuable tool for more timely diagnoses and improved patient outcomes. In summary, this study advances the frontier of ovarian cancer prediction by developing an accurate and reliable model that can be used to identify patients at high risk for the disease. This work has the potential to provide a meaningful impact on clinical practice and patient well-being, ultimately helping to save lives and improve the quality of care for those at risk of ovarian cancer. Future research and improvements for the proposed method will focus on external validation with diverse datasets, real-time implementation, and integration of longitudinal and multi-omics data. Exploring interpretable deep-learning models can enhance the method’s predictive accuracy, furthering progress in ovarian cancer diagnosis and treatment. However, Shapley graphs are not as explainable as requested in the medical domain, and other models such as PLENARY could be explored for further model explanations.

## Figures and Tables

**Table 1:** Summary of the related work.

Ref	Classifiers	Dataset	Achieved Accuracy	Biomarkers
[27]	LDA, CART, LR, NB, k-NN, SVM	Mendeley (same)	100% LR, CART, LDA	7 features
[28]	LR with LASSO	Mendeley (same)	90.6% LR with LASSO	30 features
[29]	Penalized LR/Group SCAD/Group LASSO/Group MCP, ANN, SVM	Mendeley (same)	93.33% MCP	46 feature divided into 11 groups
[30]	Proposed CNN, LDA, GoggelNetV3, Deep hybrid learning	TCGA dataset (image)	94% proposed CNN	
[31]	GMM, LR, k-NN, Bayesian linear discriminant, Non-linear regression, Detrended fluctuation analysis	E-GEOD-69207	88% GMM on DCT features	
[32]	XGB, k-NN, ADA, SVM, RF, DT	SEER	88.7% RF	
[33]	RF, GBM, LR, LGBM, DT, SVM, XGB	Mendeley (same)	91% RF, GBM, LGBM	Selected features
[12]	ROMA, DT, LR	Mendeley (same)	97.4% LR	10 features
[34]	AlexNet, VGG-16, VGG-19, GoogleNet, KK-Net proposed	TCGA dataset (image)	91% KK-Net	
[35]	RF, XGB, SVM, k-NN, NB, LR university medical centre	Tokyo women medical	80% XGB	

**Table 2:** Hyperparameter details of all classifiers.

Classifier	Hyperparameter
XGB	n_estimators = 120, learning_rate = 0.2, max_depth = 45,
RF	n_estimators = 120, criterion = ‘entropy’, max_depth = 45,
SGD	Larning_rate = ‘optimal’, epsilon = 0.2
ETC	n_estimators = 120, max_depth = 45, criterion = ‘entropy’
GBM	n_estimators = 120, learning_rate = 0.2, max_depth = 45,
KNN	n_neighbors = 5, leaf_size = 45

**Table 3:** Results of the ML models using full feature dataset.

Model	Accuracy	Precision	Recall	F1 Score
XGB	85.52	85.42	85.43	85.42
RF	85.66	86.35	87.56	86.22
SGD	84.94	85.72	85.87	85.65
ETC	86.89	86.52	86.52	86.52
GBM	87.14	87.58	87.53	87.19
KNN	77.94	77.43	78.36	77.98

**Table 4:** Results of the ensemble models.

Model	Accuracy	Precision	Recall	F1 Score
Baseline classifier (SGD + KNN)	85.94	85.67	85.56	85.61
Bagging classifier (ETC + RF)	84.93	84.63	84.45	84.76
Boosting classifier (GBM + XGB)	88.24	88.68	88.49	88.69

**Table 5:** Results of proposed ensemble model.

Model	Accuracy	Precision	Recall	F1 Score
Stacking classifier	96.87	98.84	98.92	98.88

**Table 6:** Feature importance calculated using SHAPly in descending order.

Weight	Feature	Description
0.1695 ± 0.0349	HE4	Human epididymis protein 4 is primarily associated with the female reproductive system.
0.0800 ± 0.0748	NEU	Neutrophil ratio is a numerical value that represents the ratio of neutrophils to
		Lymphocytes in a person’s blood.
0.0343 ± 0.0373	Age	Age of patient.
0.0190 ± 0.0381	CA125	Carbohydrate antigen 125 is a protein biomarker that is found in the blood.
0.0095 ± 0.0120	LYM%	Lymphocyte ratio refers to the ratio of lymphocytes to other types of white blood cells
		(leukocytes) in a person’s blood.
0.0076 ± 0.0076	CA19-9	Carbohydrate antigen 19-9 is a protein biomarker.
0.0076 ± 0.0143	ALB	Albumin is a type of protein that is found in the blood plasma.
0.0038 ± 0.0093	PCT	Thrombocytocrit is a (platelet) content of the blood.
0.0019 ± 0.0305	HGB	Hemoglobin is a protein found in red blood cells.
0.0000 ± 0.0120	AST	Aspartate aminotransferase is an enzyme found in various tissues in the body.
0.0000 ± 0.0120	GLO	Globulin is a group of proteins found in the blood plasma.
0.0000 ± 0.0000	Menopause	Menopause marks the end of a woman’s reproductive years.
−0.0019 ± 0.0076	ALP	Alkaline phosphatase is an enzyme found in various tissues throughout the body.
−0.0019 ± 0.0076	LYM#	Lymphocyte ratio or lymphocyte count.
−0.0038 ± 0.0194	CEA	Carcinoembryonic antigen is a protein.
−0.0038 ± 0.0093	PLT	Platelet count is a standard component of a complete blood count.

**Table 7:** Comparison with state-of-the-art techniques using Mendeley (same) dataset.

Ref	Proposed Classifiers	Achieved Accuracy	Biomarkers Used
[27]	LR, CART, LDA	100%	7 features
[28]	LR with LASSO	90.6%	30 features
[29]	MCP	93.33%	46 feature divided into 11 groups
[33]	RF, GBM, LGBM	91%	Selected features
[12]	LR	97.4%	10 features
[54]	RF	91%	OC marker
**Proposed**	**Stacking Classifier (bagging, boosting, Baseline)**	**96.87%**	**49 features**

**Table 8:** Results of the K-fold cross-validation during testing.

Model	Accuracy	Precision	Recall	F1 Score
1st fold	98.25	99.31	96.16	98.21
2nd fold	98.25	99.43	98.47	98.32
3rd fold	97.46	99.76	98.89	98.18
4th fold	98.80	99.87	98.76	98.85
5th fold	99.89	99.51	97.68	98.49
**Average**	**98.61**	**99.72**	**97.54**	**98.41**

**Table 9:** Results of the K-fold cross-validation during training.

Model	Accuracy	Precision	Recall	F1 Score
1st fold	99.53	99.99	99.99	99.99
2nd fold	99.67	99.96	99.98	99.97
3rd fold	99.76	99.94	99.95	99.94
4th fold	99.85	99.99	99.99	99.99
5th fold	99.78	99.97	99.96	99.96
**Average**	**99.74**	**99.97**	**99.98**	**99.98**

## Data Availability

The dataset used in this study is publicly available at the following link: https://www.kaggle.com/datasets/saurabhshahane/predict-ovarian-cancer/data, accessed on 27 October 2023.

## Appendix A. Implemented Code for SHAP

import shap

import xgboost as xgb

from sklearn.ensemble import VotingClassifier

from sklearn.linear_model import SGDClassifier

from sklearn.neighbors import KNeighborsClassifier

from sklearn.ensemble import RandomForestClassifier, ExtraTreesClassifier, GradientBoostingClassifier

xgb = xgb.XGBClassifier()

gbm = GradientBoostingClassifier(n_estimators = 100, random_state = 52)

rf = RandomForestClassifier(n_estimators = 100, random_state = 52)

etc = ExtraTreesClassifier(n_estimators = 100, random_state = 52)

sgd = SGDClassifier(max_iter = 1100, tol = 1 × 10${}^{-3}$)

knn = KNeighborsClassifier(n_neighbors = 3)

baseline = VotingClassifier(estimators = [(‘sgd’, sgd), (‘knn’, knn)], voting = ‘hard’)

bagging = VotingClassifier(estimators = [(‘etc’, etc), (‘rf’, rf)], voting = ’hard’)

boosting = VotingClassifier(estimators = [(‘gbm’, gbm), (‘xgb’, xgb)], voting = ‘hard’)

stacking = VotingClassifier(estimators = [(‘baseline’, baseline), (‘bagging’, bagging), (‘boosting’, boosting)], voting = ‘hard’)

stackingEnsemble = stacking.fit(X_train, y_train).predict(X_test)

explainer = shap.Explainer(stackingEnsemble)

shap_values = explainer(X_train)

shap.plots.beeswarm(shap_values)
